# The Influence of the Axial Rub Added in the Radial Rub on the Wear of the Seal Fins during the High Speed Rub of Labyrinth-Honeycomb Seal

**DOI:** 10.3390/ma14081997

**Published:** 2021-04-16

**Authors:** Bin Lu, Haijun Xuan, Xiaojian Ma, Fangjun Han, Weirong Hong, Shaoqiang Zhi

**Affiliations:** 1 High-Speed Rotating Machinery Laboratory, College of Energy Engineering, Zhejiang University, Hangzhou 310027, China; 11628018@zju.edu.cn (B.L.); hongwr@zju.edu.cn (W.H.); 2 Shenyang Engine Research Institute, Aero Engine Corporation of China, Shenyang 110015, China; 13842016492@163.com (X.M.); hfj307@hotmail.com (F.H.); 1zsq1@163.com (S.Z.)

**Keywords:** labyrinth-honeycomb seal, high speed rub, axial incursion

## Abstract

Labyrinth-honeycomb seals are a state-of-the-art sealing technology commonly used in aero-engine interstage seal. The undesirable severe rub between the seal fins and the honeycomb due to the clearance change may induce the cracking of the seal fins. A pervious study investigated the wear of the seal fins at different radial incursion rates. However, due to the axial thrust and mounting clearance, the axial rub between the seal fins and the honeycomb may occur. Hence, this paper focuses on the influence of the axial rub added in the radial rub on the wear of the seal fins. The rub tests results, including rubbing forces and temperature, wear rate, worn morphology, cross-sectional morphology and energy dispersive spectroscopy results, are presented and discussed. Overall, the participation of the axial rub leads to higher rubbing forces, temperature, and wear rate. The tribo-layer on the seal fin is thicker and the cracks are more obvious at high axial incursion rate. These phenomena indicate the axial rub has a negative influence on the wear of the seal fins and should be avoided.

## 1. Introduction

High efficiency and low fuel consumption have always been the goals of aero-engine design. Sealing technology is an economical and effective method to reduce gas leakage. Labyrinth seals, as non-contact seal, are widely used in the aero-engine interstage seal for its simple design, long lifetime and applicability under extreme operating conditions [1,2]. It has several teeth on the circumference of the rotor and the sealing efficiency mainly depends on the clearance between the rotor and the stator [3]. Theoretically, the smaller the clearance, the higher the efficiency. However, too small clearance may induce the severe rub between the rotor and the stator due to the clearance change caused by the thermal expansion, vibration and mechanical loading. High load and high temperature induced by the severe rub may lead to the severe wear and cracking of the seal fins. To protect the seal fins, abradable materials with low strength are commonly used as stator so that most of the wear may take place on the abradable materials during the rub. The honeycomb, as a typical abradable material, is commonly used as stator for its good erosion, corrosion, and oxidation resistances and small contact area [4].

To evaluate the abradability of the honeycomb, many researchers have attempted to improve the understanding of the rubbing behaviors between the seal fins and the honeycomb. The rub tests results in [5] showed that the aluminide coated honeycomb was more prone to stick to the seal fins during the rub and the wear of seal fins was uneven. Sporer et al. [6] executed rub tests with a knife edge ring, test blades and honeycombs made of different materials. The abradability of the honeycombs was evaluated by measuring the volume wear ratio of the test blades. The rub tests between the seal fin and the honeycomb in [7] demonstrated that the rubbing behaviors in aero-engine could be reproduced in rub tests. However there was no detailed date presented. Zhang et al. [8] published results from the rub tests between a dummy blade with two seal fins and honeycomb. They used the maximum rubbing forces to evaluate the abradability of the honeycomb at different rubbing conditions. The smearing on the worn surface of honeycomb was also observed. The Institute of Thermal Turbomachinery (ITS, Karlsruhe, Germany) has published a serious research on the rubbing behaviors between the seal fins and the honeycomb [9,10,11,12]. They first simplified the rub by replacing the honeycomb with a single metal foil. Then the rub tests with honeycomb liners were performed. The rubbing behaviors at different conditions were detailed studied. They pointed out that the insights gained using a very simple contact of a single metal foil could be transferred to the rubbing process of labyrinth seals with honeycomb liners. The rubbing behaviors were sensitive to the relatively position between the seal fins and the honeycomb. Contact forces and temperatures were much higher when the seal fins hit the double foil of the honeycomb. The analysis approaches common for turbine blade rub could be used to analyze the labyrinth seal rub. Zhang et al. [13] investigated the influence of the nickel–aluminide filler, which was filled in honeycomb, on the rubbing behaviors in a labyrinth seal system. They pointed out that the nickel–aluminide filler with high aluminum content made the abradable material easy to fracture after ageing. The fins were less worn when the abradable material was easy to fracture.

All the researches mentioned above focused on the rubbing behaviors at different rubbing conditions and the abradability of the honeycombs. The ultimate goals are to prevent wear and structural damage of the seal fins. Pychynski et al. [14] theoretically and Hühn et al. [15] experimentally identified the remaining tensile stress caused by high rubbing temperature as the main cause of cracks in the seal fins. However, the high speed rub between the seal fins and the honeycomb is a complex process including material transfer, oxidation and even microstructural changes. The seal fins should be detailly checked to improve the understanding of the wear and damage of the seal fins.

**Figure 1:**
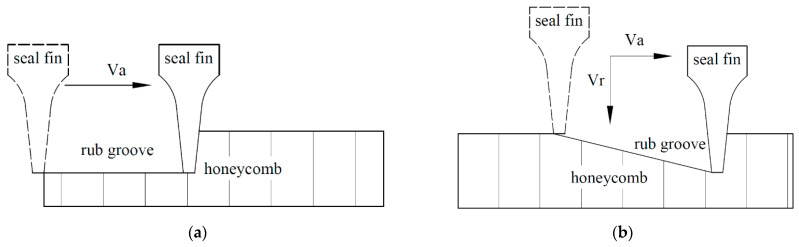
(**a**) The rubbing form in [17]; (**b**) the rubbing form in this paper. (V_a_: axial incursion rate; V_r_: radial incursion rate).

In our pervious study [16], the wear of the Ti17 seal fins during the rub between seal fin and honeycomb stator at different radial incursion rates had been investigated. The results showed that the wear mechanism of the seal fins changed from adhesive wear and oxidation wear to delamination wear and then to metal wear with the increasing radial incursion rate. Cracks appeared on the tribo-layer covered on the seal fins. The high rubbing temperature also induced the microstructural change of the seal fins. However, in aero-engine, due to the axial thrust and mounting clearance, the relative axial displacement between the seal fins and the honeycomb may occur. As a result, there is not only radial rub but also axial rub between the seal fins and the honeycomb. Axial rub tests between the seal fins and the honeycomb were performed in [17]. In their study, the seal fin with Al_2_O_3_TiO_2_ coating was in contact with aluminum–silicon coating and honeycomb in axial direction. They focused on the thermomechanical phenomena and wear flow mechanisms between the seal fins and the abradable material and proved that a hollow structure ensuring a low internal flow and a high wear flow guarantee the best operating conditions. However, the wear of the seal fins due to the axial rub were not investigated and the rubbing form (Figure 1a) was not common in aero-engine. Actually, the radial and axial rub occur simultaneously in aero-engine as shown in Figure 1b. Hence, in this paper, the rub tests between seal fins and the honeycomb with radial and axial rub are performed. The influence of the axial rub added in the radial rub on the wear of the seal fins is the focus of this paper.

## 2. Materials and Methods

### 2.1. Test Rig

**Figure 2:**
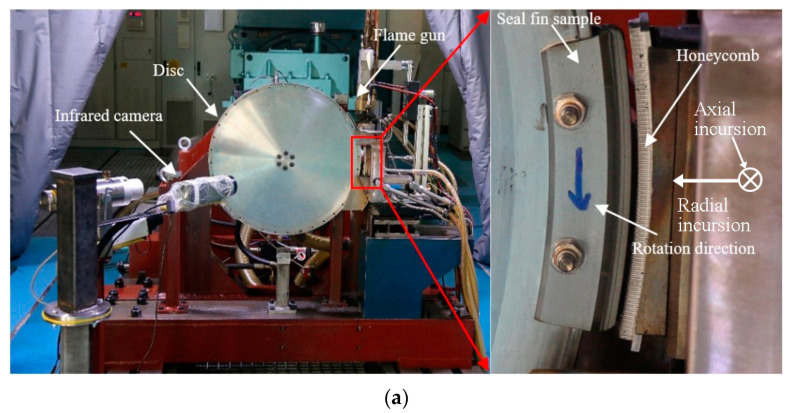

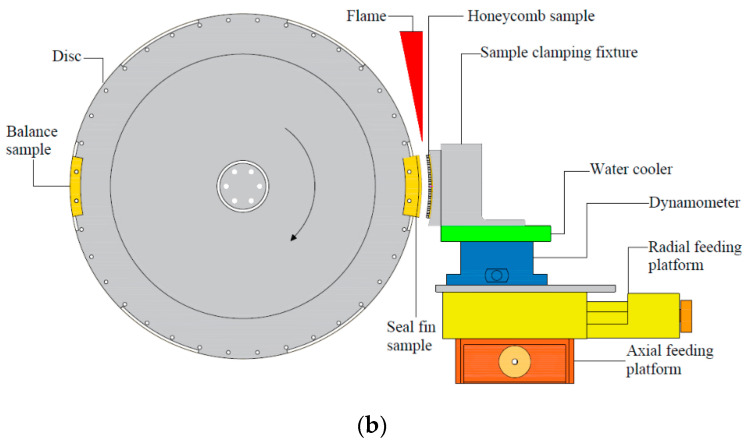
(**a**) Test rig; (**b**) test rig schematic diagram.

The test rig used was the same as that in [16] as shown in Figure 2a. In a real application, the honeycomb is static and the seal fin is moving. However, in the rub tests, the enlargement of the seal fins during rotation cannot be controlled. Hence, the rub process of the real application was equivalent to that the rotor only rotated, and the feeding platform controlled the honeycomb to move to the rotating seal fin to realize the high speed rub of the seal fins and the honeycomb. In the rub rig, the rotor was driven by the electric motor with a maximum rotor speed of 15,000 rpm. To realize the radial and axial incursion, two feeding platforms perpendicular to each other were mounted underneath the honeycomb sample as shown in Figure 2b. A flame gun using propane and oxygen as fuels was installed on the feeding platform. The temperature of flame depended on the flow rate of oxygen and propane. The flame gun could move with the feeding platform so that the relative position between the flame gun and the honeycomb did not change during the rub tests. Hence, the flame could heat the honeycomb throughout the rub tests and once the flow rates of oxygen and propane were determined, the heating temperature of the honeycomb could be kept stable. A dynamometer (Type 9257B, Kistler, Winterthur, Switzerland) was positioned underneath the honeycomb sample to measure the rubbing forces. A water cooler was mounted above the dynamometer to block the heat transfer to the dynamometer. Hence, the heat did not have great influence on the dynamometer. A high-speed acquisition device was used to collect the rubbing forces and the acquisition frequency used was 100 kHz. The instantaneous rubbing temperature on the honeycomb was measured by an infrared camera FILR A615 (FILR, Wilsonville, OR, USA) with an acquisition frequency of 100 Hz.

### 2.2. Test Samples

**Figure 3:**
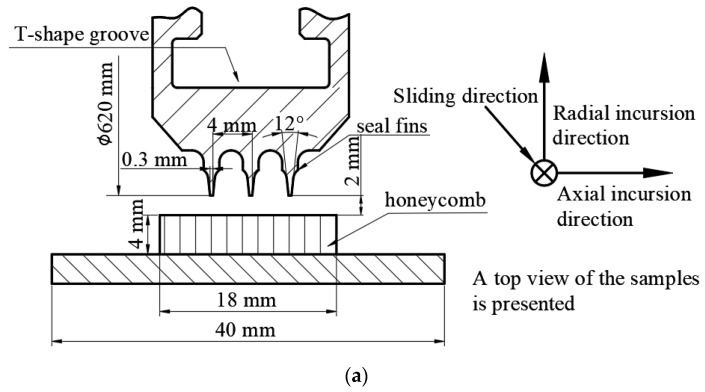

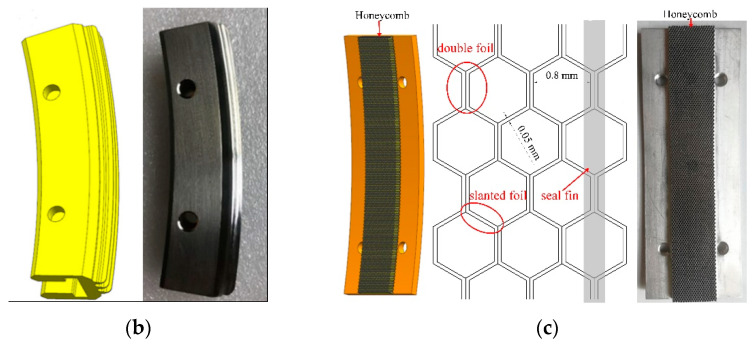
(**a**) The geometries of seal fins; (**b**) the seal fin sample and; (**c**) the honeycomb sample and honeycomb structure.

The seal fin sample and the honeycomb sample used in this paper were the same as that in [16]. The seal fin sample was an arc block with three same geometry seal fins as shown in Figure 3a. The radial, axial incursion direction and the sliding direction are also shown in Figure 3a. The diameters of the seal fins were 620 mm. As before, a seal fin ring was first manufactured and then cut into 18 seal fin samples. As a result, the angle of the seal fin sample was 20°. The objective of the design of the seal fin sample was to save the test cost and better simulate the seal fin rub form-grinding not cutting. To match the seal fins, the honeycomb sample was also an arc block with the angle of 20°. The honeycomb was formed by periodic hexagonal cell structure with the cell size of 0.8 mm. The thickness of the honeycomb foils were different: the foil parallel to the seal fin during the rub shown in Figure 3c was spot-welded together by two 0.05 mm foils. For the convenience of description, in the following text, the foil parallel to the seal fin is called double foil while the foil slanted to the seal fin is called slanted foil.

The material of the seal fin sample was Ti17 (Ti-5AI-2Sn-2Zr-4Mo-4Cr) while the honeycomb was made of Hastelloy X. These materials are commonly used in high pressure compressor. The compositions of these materials are shown in Table 1 and Table 2.

### 2.3. Test Procedure

**Figure 4:**
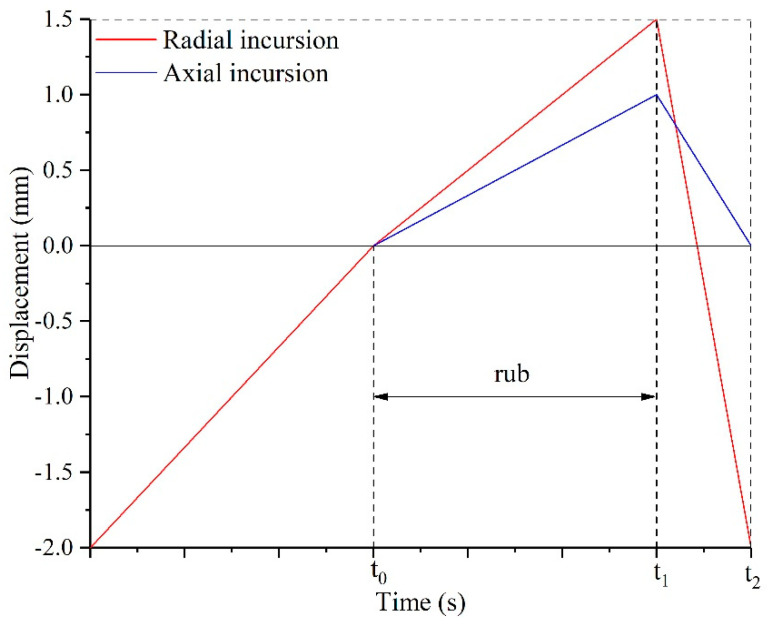
Typical incursion profile.

Before the rub tests, a temperature calibration test was performed to determinate the flow rates of oxygen and propane needed to heat the honeycomb to 350 °C. Also, the distance between the seal fin sample and the honeycomb sample was set to about 2 mm as shown in Figure 3a before the rub tests. When a rub test began, the rotor with seal fin sample driven by the electric motor was accelerated to the target speed. During the acceleration, the flame gun was ignited to heat the honeycomb. When the rotor speed and temperature were stable, the radial feeding platform first pushed the honeycomb to the seal fins in radial direction at an incursion rate of 50 μm/s and the axial feeding platform remained stationary. Once the seal fins were in contact with the honeycomb, the radial feeding platform and the axial feeding platform pushed the honeycomb sample to move in radial and axial direction simultaneously at preset radial and axial incursion rate. Once the radial incursion depth was reached, the radial and axial feeding platform retracted immediately. A typical incursion profile for a rub test is shown in Figure 4. The rubbing forces and temperature were recorded simultaneously. After one rub test, the seal fin sample and the honeycomb sample were replaced by new samples so that the wear of the seal fins, which were checked by scanning electron microscope (SEM, Zeiss, Oberkochen, Germany) and energy dispersive spectroscopy (EDS, Zeiss, Oberkochen, Germany), corresponded to the axial incursion rates one by one. The mass change of the seal fins was measured by an electronic balance with an accuracy of 1 mg before and after the rub test to calculate the wear rate of the seal fins.

### 2.4. Test Parameters

In the rub tests, the rubbing speed, temperature, radial incursion rate, and radial incursion depth were kept constant. The research focused on the influence of axial incursion rate on the wear of the seal fins. The test matrix is shown in Table 3. The temperature and rubbing speed were the same as the rub conditions in the high pressure compressor. In a real application, the combined radial and axial rub are acting just on the short term. The radial incursion rate and radial incursion depth were set to 100 μm/s and 1500 μm respectively. Hence the rubbing time was 15 s. The axial incursion rates were set from 60 μm/s to 120 μm/s with the interval of 20 μm/s. Due to the constant rubbing time, the axial incursion depths were different as listed in Table 3.

## 3. Results

For the convenience of comparison, the rub test results (rubbing forces and temperature, wear rate, worn morphology and EDS results) at radial incursion rate of 100 μm/s without axial rub are cited from [16] (test no. I-3) and presented here. Since the radial incursion rate was constant, the incursion rate refers to the axial incursion rate in the following if there is no special description.

### 3.1. The Rubbing Forces, Temperature, and the Wear Rate at Different Axial Incursion Rates

**Figure 5:**
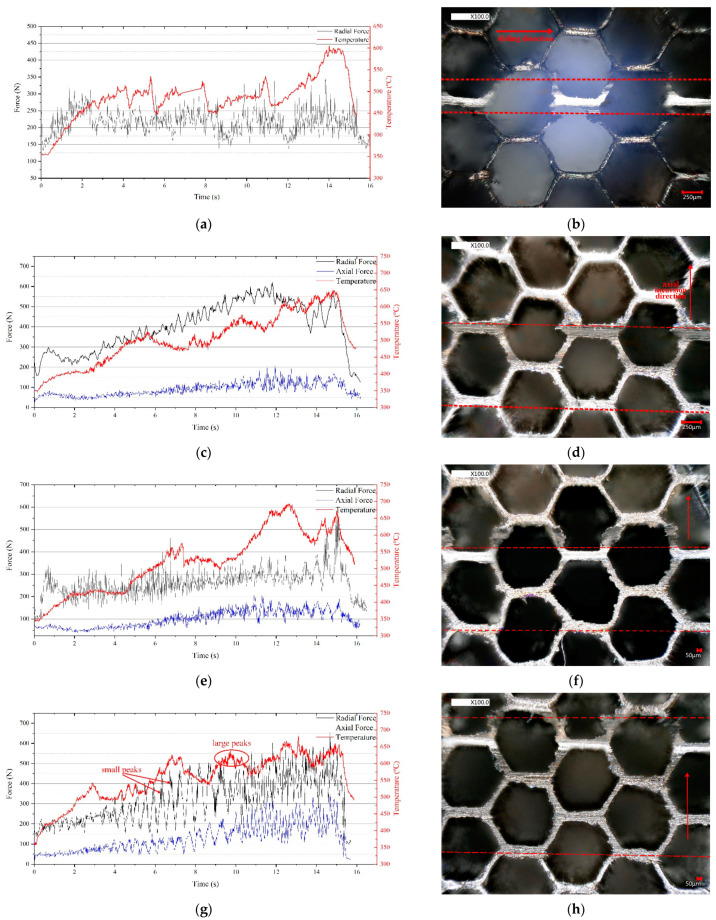

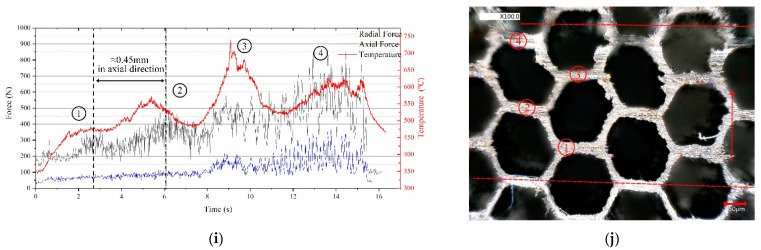
The rubbing forces and temperature and the wear grooves of the honeycomb at different axial incursion rates; (**a**,**b**) only radial rub; (**c**,**d**) 60 µm/s; (**e**,**f**) 80 µm/s; (**g**,**h**) 100 µm/s; (**i**,**j**) 120 µm/s; (**a**) is cited from reference [16].

The radial and axial rubbing forces and the rubbing temperature at different axial incursion rates are shown in Figure 5a,c,e,i,j. The rubbing forces curves are envelopes extracted from the original data to make the images more concise.

**Figure 6:**
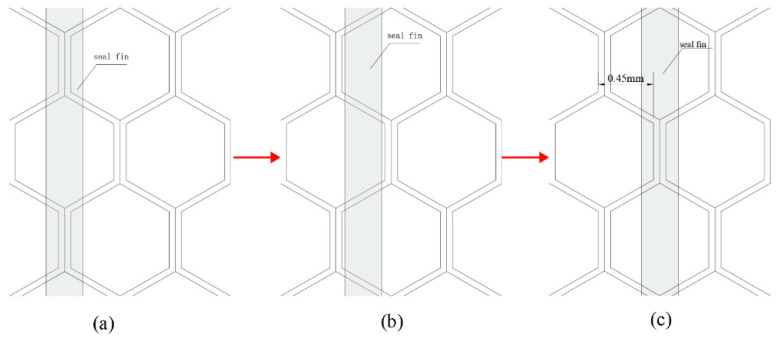
(**a**–**c**) The rub position between the seal fin and the honeycomb during axial rub.

The axial force was significantly lower than the radial force. The radial and axial forces were prone to increase with the incursion depth. This was most likely due to the increasing contact area [12]. When there was axial rub, large peaks appeared on the curves of the rubbing forces and temperature, which were mainly caused by the different rub positions of the seal fins and the honeycomb. There were two rub positions as shown in Figure 6. The rubbing forces and temperature were lower when the seal fins hit the slanted foil for its small contact area, which was also proved in [12,17]. The distance between the two adjacent double foil was 0.45 mm as shown in Figure 6c. As a result, the rubbing forces and the temperature should have a large peak per 0.45 mm in axial incursion direction. However, this regular was only obvious at 120 μm/s. The peaks 1, 2, 3, and 4 in Figure 5i corresponded to the double foil 1, 2, 3, and 4 in Figure 5j. At other axial incursion rates, this regular was not obvious. The reason may be that the seal fin sample had three seal fins which may not hit the double foil simultaneously due to the mounting error. When there was only radial rub as shown in Figure 5b, the rub position between the seal fin and the honeycomb was not changed. The large peaks in Figure 5a were caused by the periodical delamination of the tribo-layer on the seal fin as discussed in [16]. 

Apart from the large peaks, the small peaks on the curves of rubbing forces and temperature were also obvious when there is axial rub. With the increasing axial incursion rate and the incursion depth, these small peaks were more obvious. The reason to explain this phenomenon was that the increasing axial incursion rate and the incursion depth increased the wear of the honeycomb, which provided sufficient debris promoting the generation rate of the tribo-layer. Hence, the period of the generation and spalling of the tribo-layer was shorter and the large peaks when there was only radial rub became the small peaks when there was axial rub. The tribo-layer on the seal fins will be presented in Section 3.3 and discussed in Section 4.

**Figure 7:**
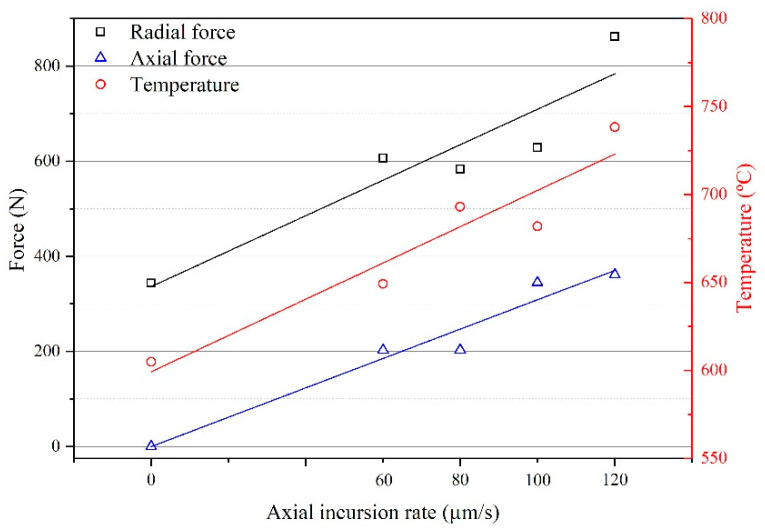
The maximum rubbing forces and temperature at different axial incursion rates.

The maximum radial and axial rubbing forces and temperature at different axial incursion rates are shown in Figure 7. It can be clearly seen that the maximum radial and axial rubbing force and temperature present an increasing trend with the axial incursion rate.

**Figure 8:**
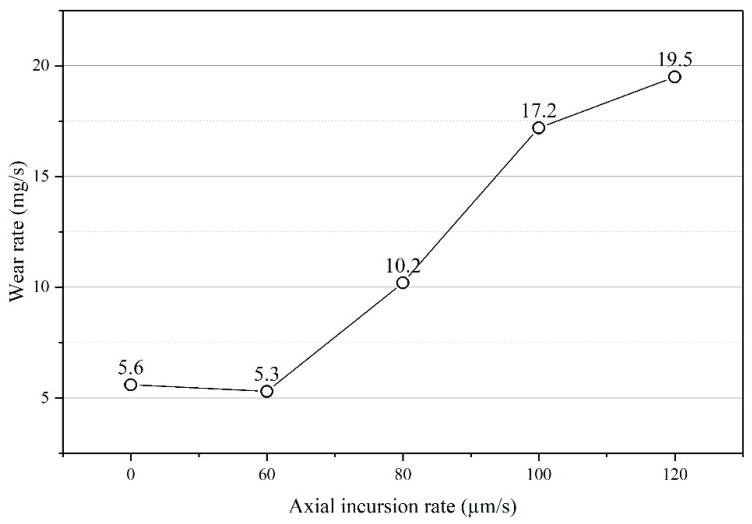
The wear rate of the seal fin at different axial incursion rates.

The wear rate here is defined as the wear loss of the seal fins per second. The wear rate of the seal fins at different axial incursion rates is shown in Figure 8. At 60 μm/s, the wear rate is slightly lower than that with only radial rub. After the 60 μm/s, with the increasing axial incursion rate, the wear rate increases monotonously.

### 3.2. The Worn Morphology of the Seal Fins

**Figure 9:**
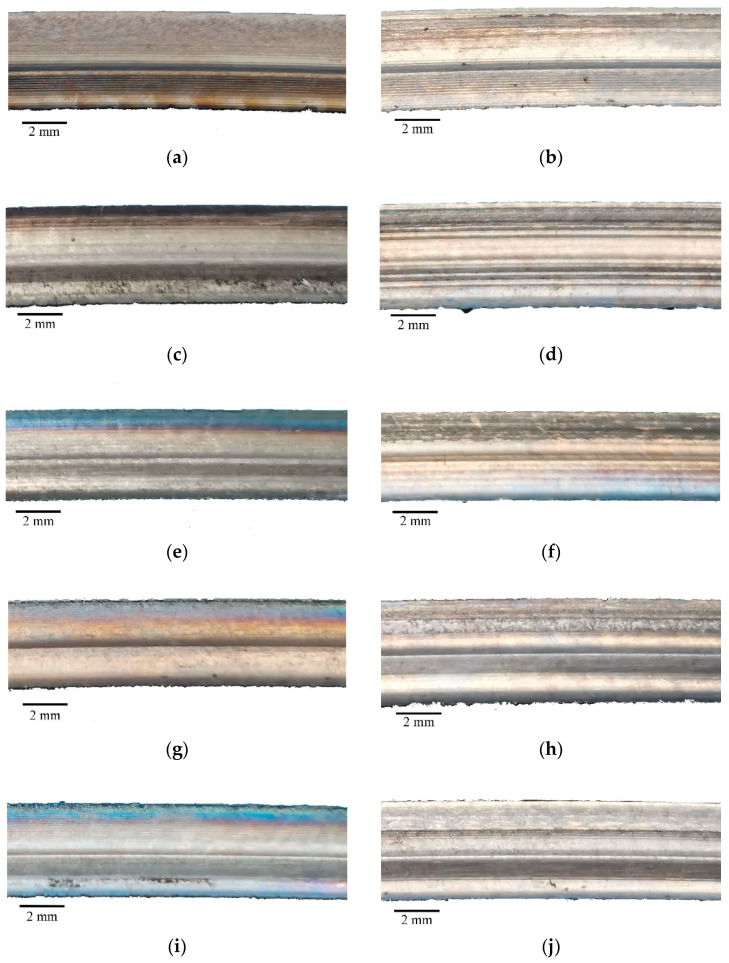

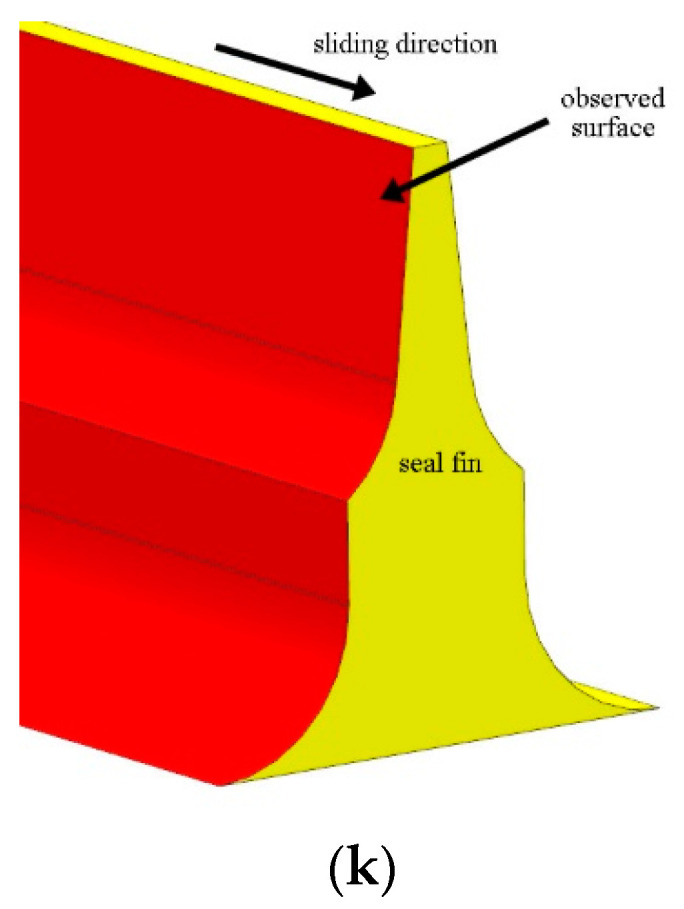
The macroscopic morphology of the seal fin. (**a**) The original seal fin; (**b**) the seal fin with only radial rub; (**c**,**e**,**g**,**i**) the axial rub sides of the seal fins at 60, 80, 100, and 120 μm/s respectively; (**d**,**f**,**h**,**j**) the non-axial rub sides of the seal fins at 60, 80, 100, and 120 μm/s respectively; (**k**) the observed surface.

The macroscopic morphology of the side of the seal fin is shown in Figure 9. Figure 9a is the original seal fin and Figure 9b is the seal fin with only radial rub. The morphologies of two sides of the seal fins are similar. Hence, only one side of the seal fin is presented. Figure 9c,e,g,i present the axial rub side, while Figure 9d,f,h,j present the non-axial rub side. It can be clearly seen that the discoloration on the axial rub side is obvious with the increasing axial incursion rate. The discoloration does not appear on the non-axial rub side of the seal fin. The discoloration of the seal fin indicates that the rubbing temperature increases with axial incursion rate. The temperature on the axial rub side is much higher than that on non-axial rub side.

**Figure 10:**
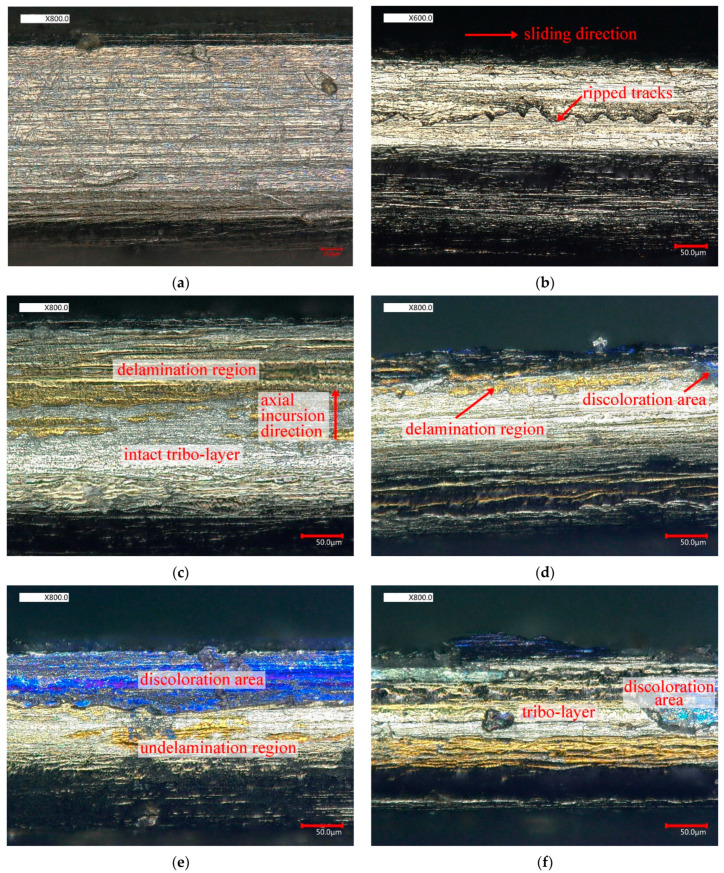
The worn morphology of the seal fin observed by ultra-depth-of-field microscope. (**a**) the original seal fin; (**b**) only radial rub; (**c**) 60 μm/s; (**d**) 80 μm/s; (**e**) 100 μm/s; (**f**) 120 μm/s.

**Figure 11:**
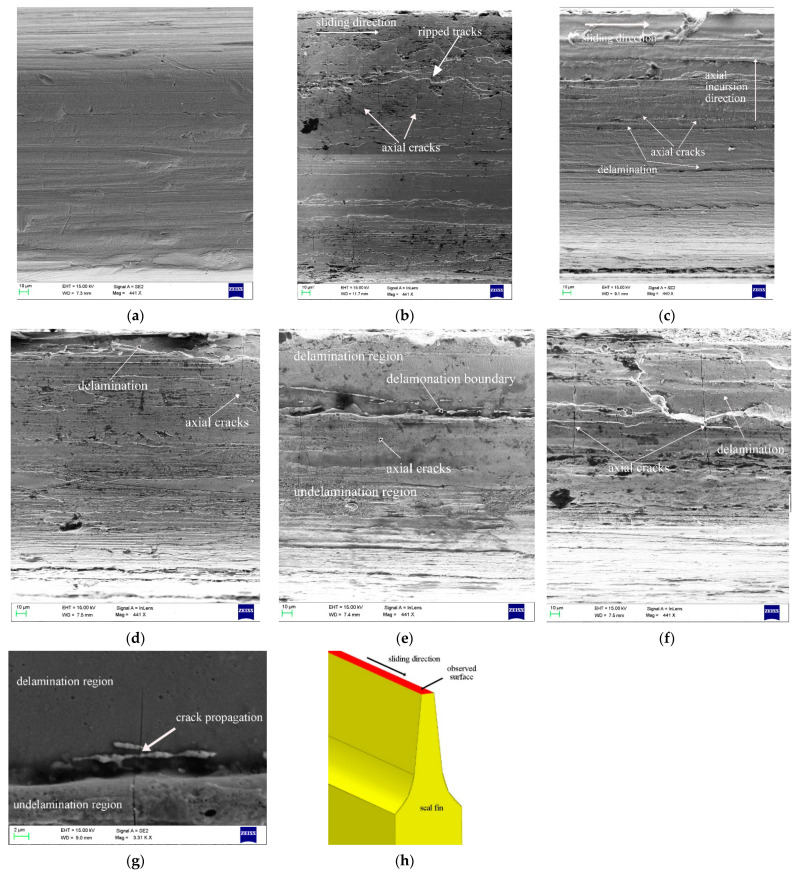
The worn morphology of the seal fin observed by SEM. (**a**) The original seal fin; (**b**) 0 μm/s; (**c**) 60 μm/s; (**d**) 80 μm/s; (**e**) 100 μm/s; (**f**) 120 μm/s; (**g**) the partial enlargement figure of (**e**); (**h**) the observed surface; (**a**,**b**) are cited from reference [16].

The worn morphology of the top surface of the seal fin observed by ultra-depth-of-field microscope is shown in Figure 10. The observed surface is the red surface shown in Figure 11h. The red arrows in Figure 10b,c indicate the sliding direction and axial incursion direction respectively. Compared with the original seal fin as shown in Figure 10a, the white tribo-layer with ripped tracks covered the worn surface as shown in Figure 10b. With the participation of the axial rub, at 60 and 80 µm/s, same regions are golden and some regions are white. It can be clearly seen that the delamination occurs on the golden regions. At 100 and 120 µm/s, extensive delamination of the tribo-layer occurs and the delamination regions are blue-purple. The delamination and spalling of the tribo-layer exposes the Ti17 substrate, which is ablated and discolored under high rubbing temperature. The golden regions and blue-purple regions are more obvious near the axial rub side proving the rubbing forces and temperature are high near the axial rub side. Also, the transition from the golden regions at low axial incursion rate to the blue-purple regions at high axial incursion rate indicates that the rubbing temperature is higher at high axial incursion rate.

The worn morphology of the top surface of the seal fin observed by SEM is shown in Figure 11. The white arrows in Figure 11b and c indicate the sliding direction and axial incursion direction respectively. In SEM images, the cracks perpendicular to the sliding direction can be clearly identified. These cracks are called axial cracks here. When there is only radial rub as shown in Figure 11b, the cracks only appear on the edges of the seal fin. At 60 μm/s and 80 μm/s, the axial cracks run through the entire surface from one side to another side. The cracks are relatively narrow and shallow. At 100 μm/s, the axial cracks appear on the undelamination region, while the axial cracks disappear on the delamination region. However, at the boundary of delamination region and undelamination region, the axial cracks propagate from the undelamination region to the delamination region as shown in Figure 11g. This phenomenon proves that the cracks on the tribo-layer may induce the cracking of the substrate. At 120 μm/s, the axial cracks are wide and deep and run through the entire surface. The axial cracks at delamination region are also obvious.

### 3.3. The Cross-Sectional Morphology of the Seal Fin

**Figure 12:**
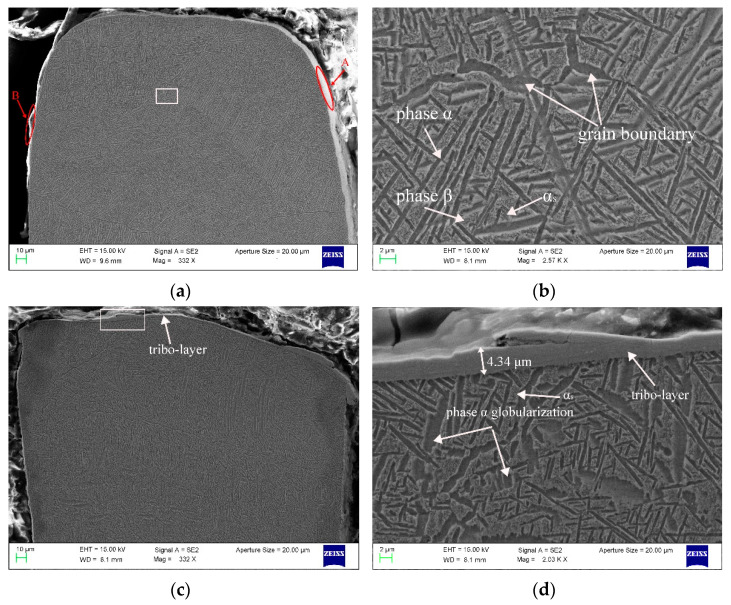

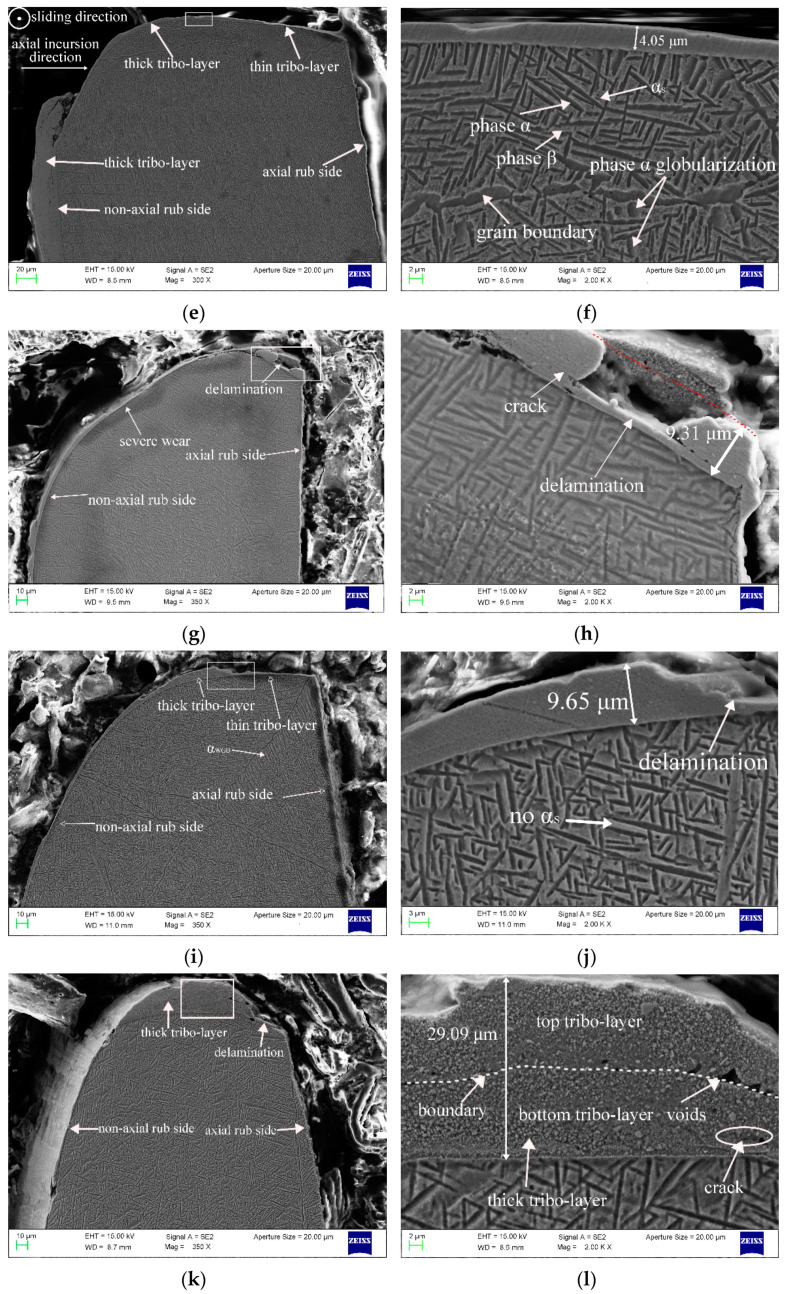

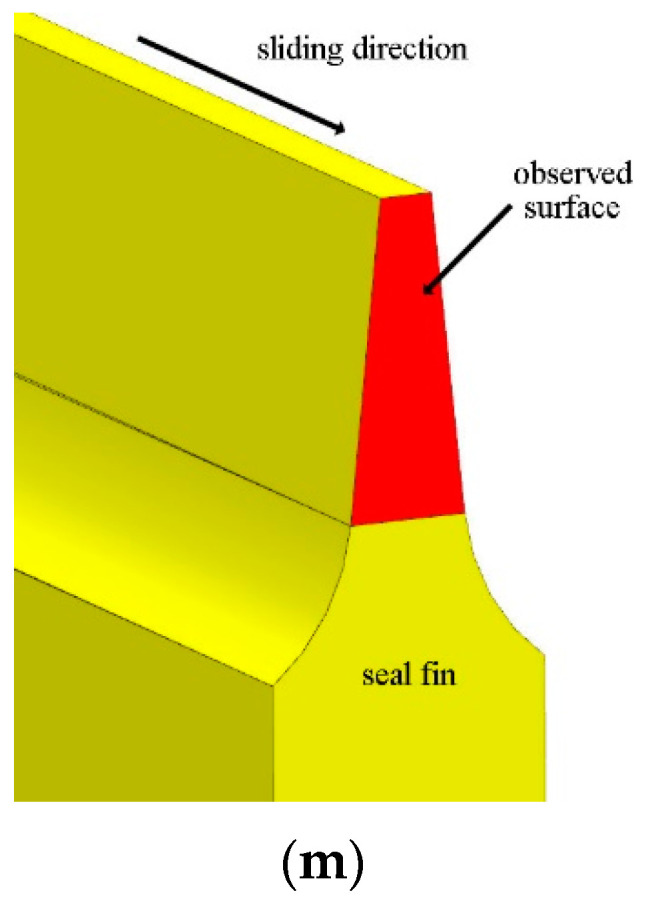
The cross-section of the seal fin. (**a**,**b**) The original seal fin; (**c**,**d**) only radial rub; (**e**,**f**) 60 μm/s; (**g**,**h**) 80 μm/s; (**i**,**j**) 100 μm/s; (**k**,**l**) 120 μm/s; (**m**) the observed surface; (**a**,**c**) are cited from reference [16].

The seal fin was cut perpendicular to the sliding direction to observe the cross-section (red surface in Figure 12m). The cross-sectional morphology of the seal fin is shown in Figure 12. The white arrow and the white circle in Figure 12e indicate the axial incursion direction and the sliding direction respectively. There is no tribo-layer on the original seal fin as shown in Figure 12a. It is to be noted that the bright edges in region A as shown in Figure 12a are the materials behind the surface because the specimen is slightly tilted. The region B in Figure 12a is the inlay rather than the tribo-layer. The wear of the both sides of the seal fin is uniform and the wear is slight when there is only radial rub as shown in Figure 12c. It can be clearly seen that the wear on the axial rub side is severe and the wear on the non-axial rub side is slight when there is axial rub as shown in Figure 12e,i,j,k. The tribo-layer only covers the top surface of the seal fin when there is only radial rub. However, the tribo-layer covers both the top surface and the non-axial rub side when there is axial rub. The reason for the retention of the tribo-layer on the non-axial rub side can be attributed to the low rubbing forces and temperature on the non-axial rub side. On the axial rub side, no tribo-layer appears. The maximum thickness of the tribo-layer on the top surface of the seal fin is 4.34, 4.05, 9.31, 9.65, 29.09 μm at 0, 60, 80, 100, 120 μm/s respectively, as shown in Figure 12d,f,g,h,l. Except that the thickness of tribo-layer at 60 μm/s is less than that with only radial rub, the thickness of tribo-layer at other axial incursion rates is thicker than that with only radial rub and the tribo-layer thickness increases with the axial incursion rate. However, the tribo-layer is not a whole as shown in Figure 12l. The tribo-layer can be divided into two parts: the top tribo-layer and the bottom tribo-layer. The voids appear between the top tribo-layer and the bottom tribo-layer, which indicates that the top tribo-layer and the bottom tribo-layer are not firmly adhered together. Relatively, the adhesion between the bottom tribo-layer and the substrate is firm. As a result, the top tribo-layer will be easily peeled off during the rub while the bottom tribo-layer is hard to be peeled off and retains on the seal fin. However, cracks still exist in the bottom tribo-layer as shown in the white circle in Figure 12l, which indicates that the bottom tribo-layer also may be peeled off under high load. It is to be noted that the initial contour of the tribo-layer is the red dotted line in the Figure 12h. The spalling of tribo-layer is caused by grinding during metallographic sample preparation. This also proves that the top tribo-layer is easily peeled off. 

The original microstructure of the seal fin shows basket-weave microstructure with tiny acicular secondary α phase (α_s_) in β phase as shown in Figure 12b. When there is only radial rub as shown in Figure 12d, the substrate presents globularization of α phase due to the high rubbing temperature and periodic thermal cycle which is discussed in [16]. The α_s_ still exists in the β phase. At 60 μm/s, the degree of α phase globularization, as shown in Figure 12f, is smaller than that in Figure 12d. The α_s_ becomes blurred which indicates that the α_s_ begins to dissolve in β phase. For Ti17, the transition temperature from α phase to β phase is between 660 °C to 905 °C [18]. As shown in Figure 7, the maximum rubbing temperature at 60 μm/s is near 660 °C. Hence, the transition from α phase to β phase begins. According to the research in [19], α_s_ will first dissolve in β phase for titanium alloy. Hence the α_s_ becomes blurred at 60 μm/s. With the increasing axial incursion rate, the rubbing temperature increases resulting in the completely dissolution of α_s_ as shown in Figure 12h,j,l. At 100 μm/s, the microstructure at the grain boundary near the axial rub side presents a special α phase colony as shown in Figure 12i. This α phase is called α_WGB_ (Widmanstätten grain boundary). The α_WGB_ systematically grows from α_GB_ (grain boundary) and is composed of colonies of parallel α plates with often only one crystallographic orientation [20]. The appearance of the α_WGB_ indicates the recrystallization of α phase. Aeby-Gautier et al. [21] pointed out that when the transformation temperature is between 700 and 740 °C, the relative amounts of the α_WGB_ and α_WI_ (α phase in this paper) are similar. Hence, rubbing temperature may be between 700 and 740 °C. According to Figure 7, the maximum rubbing temperature at 100 μm/s is 682 °C, which is lower than 700 °C. However, according to the fitted curve of the temperature, the maximum temperature is 702 °C, which is between 700 and 740 °C. The lower test data may be attributed to the test error. Compared with the Figure 10e and Figure 12j, the region with α_WGB_ is covered by thin tribo-layer with a thickness of only about 0.5 μm and this region presents blue-purple color. These features indicate that the thick tribo-layer may have an insulating effect. At 120 μm/s, the α_WGB_ does not exist in substrate microstructure although the maximum rubbing temperature is higher. This may be attributed to the insulating effect of the thick tribo-layer as shown in Figure 12l.

### 3.4. EDS Results

**Figure 13:**
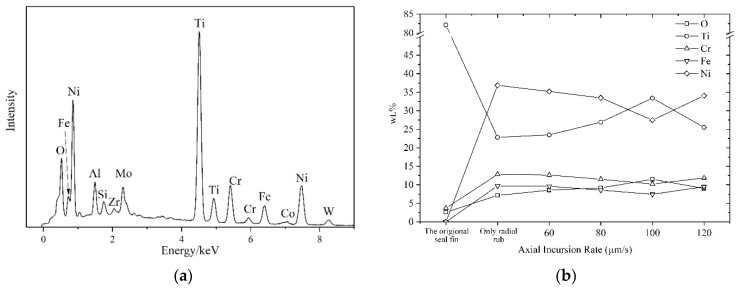
(**a**) The EDS spectrum at 100 μm/s; (**b**) the weight percentage of five elements at different incursion rates.

The EDS results of the top surface of the seal fin are shown in Figure 13. The EDS-spectra at different incursion rates is similar. Hence only EDS-spectra at 100 μm/s is shown in Figure 13a. The O, Ti, Cr, Fe, Ni are five main elements. The wt.% (weight percentage) of these five elements at different axial incursion rates is shown in Figure 13b. The original seal fin does not have Fe and Ni. Similar to the radial rub, the participation of axial rub does not change the composition of the tribo-layer. The wt.% of the five main elements at different axial incursion rats are similar. Only at 100 μm/s, the wt.% of Ti and O are higher and the wt.% of Ni, Cr, Fe are lower which may be due to the delamination of the tribo-layer near the axial rub side as shown in Figure 12i. The Ni, Cr, and Fe are elements in honeycomb which indicates that some of the materials in honeycomb transferred to the seal fin during the rub.

**Figure 14:**
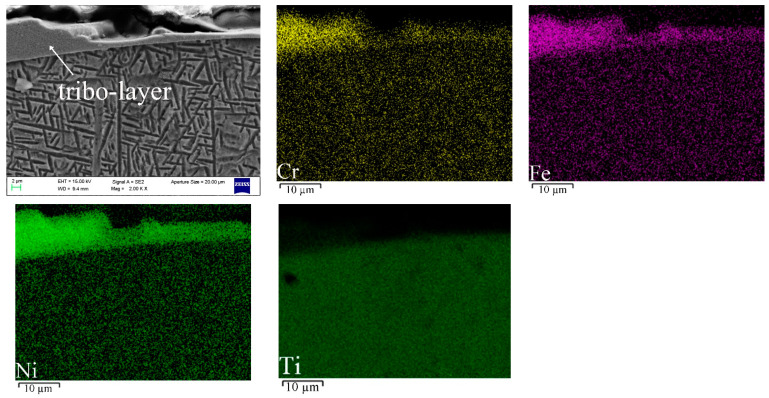
The EDS mappings of the typical cross-sectional surface of seal fin (100 µm/s).

The EDS mappings of the typical cross-sectional surface of seal fin are shown in Figure 14. It can be clearly seen that the concentration of Cr, Fe and Ni in the tribo-layer, while the content of Ti in tribo-layer is low. The distribution of these elements proves the existence of the tribo-layer.

## 4. Discussion

**Figure 15:**
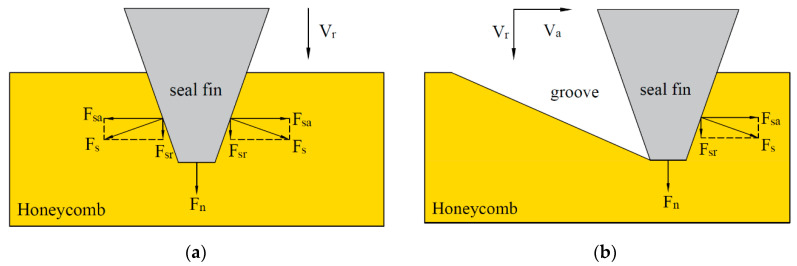
The generation of the rubbing forces; (**a**) radial rub; (**b**) radial rub and axial rub.

With the increasing radial incursion depth, the contact area between the side surface of the seal fin and the honeycomb increases as shown in Figure 15, leading to the increase of the side contact forces (F_s_). When there is only radial rub, the F_sr_ decomposed from F_s_ increases, leading to the increase of the total radial force. While the two F_sa_ decomposed from two F_s_ are equal in magnitude and opposite in direction, so they cancel each other as shown in Figure 15a. With the participation of the axial rub, the decomposed F_sa_ cannot be cancelled so that the axial force appears as shown in Figure 15b. With the increasing axial incursion rate, the axial incursion depth per revolution increases, leading to the increase of the rubbing forces. Accomplished by the increasing rubbing force, the rubbing temperature also increases. It is to be noted that, due to the periodical structure of the honeycomb, the large forces and temperature only appear when the seal fins hit the double foil.

According to the worn surface morphology and cross-sectional morphology and EDS results, the wear mechanism of the seal fin at different axial incursion rates can be analyzed. On axial rub side of the seal fin, there is no tribo-layer which indicates that the metal wear is the dominated wear mechanism. On the top surface of the seal fin, the worn surface presents the delamination of the tribo-layer. Hence, the delamination wear is the dominated wear mechanism. The participation of the axial rub does not change the wear mechanism.

**Figure 16:**
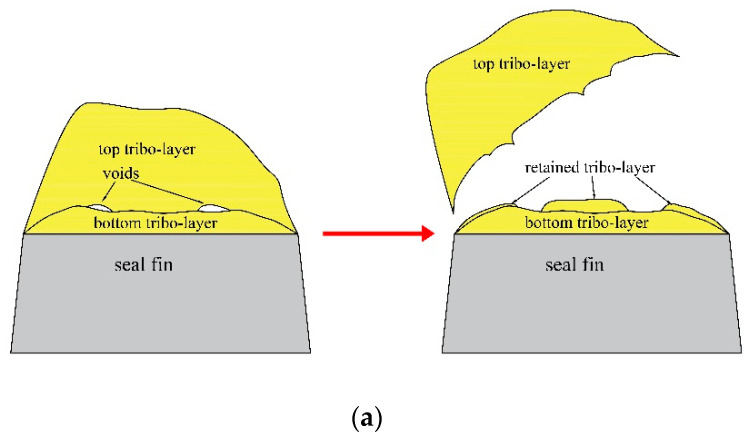

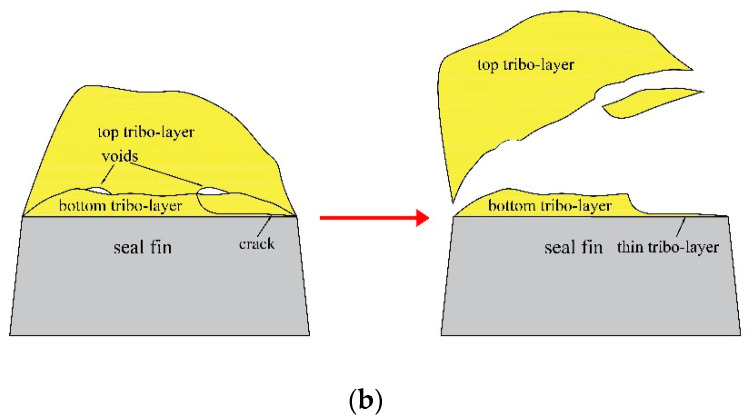
(**a**) The thickening of the tribo-layer; (**b**) the formation of the thin tribo-layer.

As discussed in Section 3.3, the tribo-layer can be divided into two parts: the top tribo-layer and the bottom tribo-layer. This is especially true at 120 µm/s as shown in Figure 12l. The top tribo-layer is easily peeled off under high load and temperature during the rub. However, some of the top tribo-layer may retain and blend with the bottom tribo-layer. This process resulting in the thickening of the tribo-layer as the rub proceeds as shown in Figure 16a. However, there is another situation as shown in Figure 16b. If the bottom tribo-layer is not compact, as shown in Figure 12l, where the cracks appear in the bottom tribo-layer, some of the bottom tribo-layer may also be peeled off leading to the thin tribo-layer on the seal fin as shown in Figure 12i,k. 

Accompanied by periodical formation and spalling of the tribo-layer, small peaks on the curves of rubbing force and temperature become obvious. In the test results in [9,10,11], the small peaks on the rubbing forces and temperature curves were not as obvious as that as shown in Figure 5. However, these small peaks were more obvious in the tests results in [12], where the single metal foil was replaced by honeycomb liner with the angle of 7.2°. It can be deduced that the wear of the large honeycomb liner provided more wear debris than the single metal foil and promoted the formation of the tribo-layer. In this paper, the test temperature of 350 °C softened the material and facilitated the wear debris to stick to the seal fins to form the tribo-layer. Also, the wear of the seal fins in this paper was noticeable, which facilitated the spalling of the tribo-layer, while the wear of the seal fins in [9,10,11,12] was negligible. Hence, the honeycomb used in the rub tests should be large enough and the test temperature should be similar to that in the aero-engine to better simulate the formation of the tribo-layer and reflect the fluctuation of the rubbing forces and temperature.

Combined with the Figure 9, Figure 10 and Figure 12, it can be clearly seen that the seal fin is discolored in the regions where there is thin tribo-layer or no tribo-layer. The discolored regions indicate that the high rubbing temperature has a great influence on the seal fins. The high rubbing temperature exceeds the phase transition temperature and induces α phase globalization, α_s_ dissolution, and even α phase recrystallization as shown in Figure 12. The mechanical properties of Ti17 highly depend on the microstructure. The participation of acicular α_s_ will increase the microhardness [22] and tensile strength and yield strength [23]. The acicular microstructure also provides high threshold stress intensity factor range and high fatigue crack growth resistance [24]. The dissolution of acicular α_s_ may be deleterious to these properties. The globalization of α phase has a negative influence on the impact toughness of Ti17 alloy and makes it easier for the initiation and propagation of cracks [25]. Due to the decrease of the properties of the seal fins, the cracks generated during the rub may propagate rapidly during the operation of the aero-engine, which will eventually reduce the service life of the aero-engine and threaten the safety of the aero-engine.

The regions of the seal fin that are covered by thick tribo-layer are not discolored and α phase recrystallization does not occur. This is particularly true at 120 μm/s with thick tribo-layer as shown in Figure 12k. Hence, it can be concluded that the thick tribo-layer can block the heat transfer to the substrate and protect the seal fin from microstructural change. However, as shown in Figure 11f, the thick tribo-layer is easy to crack to form wide and deep axial cracks. These axial cracks propagation may induce the cracking of the seal fin substrate. Therefore, it is unrealistic to reduce the influence of the high rubbing temperature on the seal fin by forming a thick tribo-layer.

In a sum, the participation of the axial rub will increase wear rate of the seal fin and produce wide and deep axial cracks by generating thick tribo-layer. The high rubbing temperature also induces the microstructural change on the substrate. As a result, the axial rub added in the radial rub has a negative influence on the wear of the seal fins.

## 5. Conclusions

This paper investigates the influence of the axial rub on the wear of the seal fins through the rub tests between the labyrinth seal fins and the honeycomb with radial and axial rub. It is shown that the axial rub added in the radial rub will have a negative influence on the wear of the seal fin, especially at higher axial incursion rate. The detailed conclusions are as follows:

1. The maximum rubbing radial force, axial force, and the temperature increase with the axial incursion rate. When the axial incursion rate is high (80, 100, 120 μm/s), the wear rate of the seal fins is higher than that when there is only radial rub and the wear rate continuously increases with the axial incursion rate.
2. When the radial incursion rate keeps constant, the axial rub will not change the wear mechanism. The delamination wear is dominated on the top surface of the seal fin, while the metal wear is dominated on the axial rub side of the seal fin.
3. The tribo-layer on the top surface of the seal fin will thicken with the axial incursion rate. The thick tribo-layer can block the heat transfer to the substrate. However, the thick tribo-layer is more prone to crack under periodical thermomechanical stress and the cracks are wide and deep.
4. When the axial incursion rate is low (60 μm/s), slight α phase globularization and dissolution of secondary α phase occur on the substrate microstructure. With the increasing axial incursion rate, the increasing rubbing temperature will induce the complete dissolution of secondary α phase and even the recrystallization of the α phase. The microstructural change of the seal fins will reduce the strength and the cracking resistance of the seal fins, which may lead to the rapid propagation of the cracks during the operation of the aero-engine.

## Figures and Tables

**Table 1:** The compositions of Ti17 alloy (wt.%).

Composition	Ti	Al	Cr	Zr	Mo	Sn
Content/%	balance	5.03	3.88	1.99	4.02	2.07

**Table 2:** The compositions of Hastelloy X alloy (wt.%).

Composition	Ni	Cr	Fe	Mo	Co	Al
Content/%	balance	21.74	19.18	8.46	1.31	0.13
Composition	W	Si	C	P	Cu	-
Content/%	0.65	0.24	0.066	0.014	0.08	-

**Table 3:** Test parameters.

Test No.	Axial Incursion Rate (V_ainc_) (μm/s)	Axial Incursion Depth (D_a_) (μm)	Radial Incursion Rate (V_rinc_) (μm/s)	Radial Incursion Depth (D_r_) (μm)	Temperature (T) (°C)	Rubbing Speed (V_t_) (m/s)
V-1	60	900	100	1500	350	380
V-2	80	1200				
V-3	100	1500				
V-4	120	1800				

## Data Availability

Data sharing not applicable.
